# Langerhans cell histiocytosis in an adult patient with upper jaw and pulmonary involvement: A case report

**DOI:** 10.1515/biol-2022-1022

**Published:** 2025-03-28

**Authors:** Yingjie Xiong, Fei Xu

**Affiliations:** Department of Stomatology, People’s Hospital of Longhua, Shenzhen, 518109, People’s Republic of China; Department of Stomatology, Xiangya Hospital Central South University, No. 87 Xiangya Road, Kaifu District, Changsha, Hunan, 410008, China

**Keywords:** Langerhans cell histiocytosis, oral cavity, pulmonary involvement, case report

## Abstract

Langerhans cell histiocytosis (LCH) is characterized by proliferative histiocyte-like cells. LCH lesions exhibit diverse and nonspecific clinical features, often leading to misdiagnosis and delayed treatment. Primary LCH lesions in the oral cavity remain rare. We report a case of a 27-year-old man with rare adult-onset LCH presenting with progressive mobility of the posterior teeth. The patient experienced increasing tooth mobility and subsequent loss in the upper and lower jaw despite undergoing periodontal treatment. A biopsy of oral mucosal tissue confirmed the Langerhans cell origin. High-resolution computed tomography imaging revealed pulmonary involvement. After undergoing systemic chemotherapy with prednisolone, vinblastine, and etoposide, the patient exhibited favourable follow-up outcomes. This case underscores the value of early diagnosis to prevent disease progression and highlights the need for dentists to be aware of LCH.

## 1 Introduction

Langerhans cell histiocytosis (LCH) is a rare disorder characterized by the accumulation of pathological dendritic histiocytes known as Langerhans cells. It can manifest at any age but is more common in children [1,2]. The clinical presentation of LCH varies widely, from mild cases with self-healing solitary lesions to severe multisystem disease with potentially fatal dissemination. Localized disease is classified as single-system LCH (SS-LCH), whereas disease affecting multiple systems is classified as multisystem LCH (MS-LCH). SS-LCH accounts for over 60% of cases and primarily affects the bone, skin, and lymph nodes, whereas MS-LCH involves organs such as the liver, spleen, and bone marrow [3].

LCH exhibits a notable predilection for the head and neck region, with reported morbidity rates ranging from 55 to 80% [4]. Clinically, oral manifestations of LCH can affect various structures, presenting as osteolytic lesions, pathological jaw fractures, masses, gingival inflammation or recession, excessive tooth mobility or loss, malocclusion, and ulceration [5,6,7]. Specifically, osteolytic lesions tend to more frequently affect the mandibular molar region compared with the maxillary region [8]. However, primary lesions within the oral cavity are relatively uncommon. Among oral soft tissues, the gingiva and hard palate mucosa are most involved [9]. We report a case of an adult with LCH, involving the upper jaw and pulmonary regions, and summarize the clinical manifestations, histopathologic characteristics, and differential diagnosis.

## 2 Case presentation

**Figure 1:**
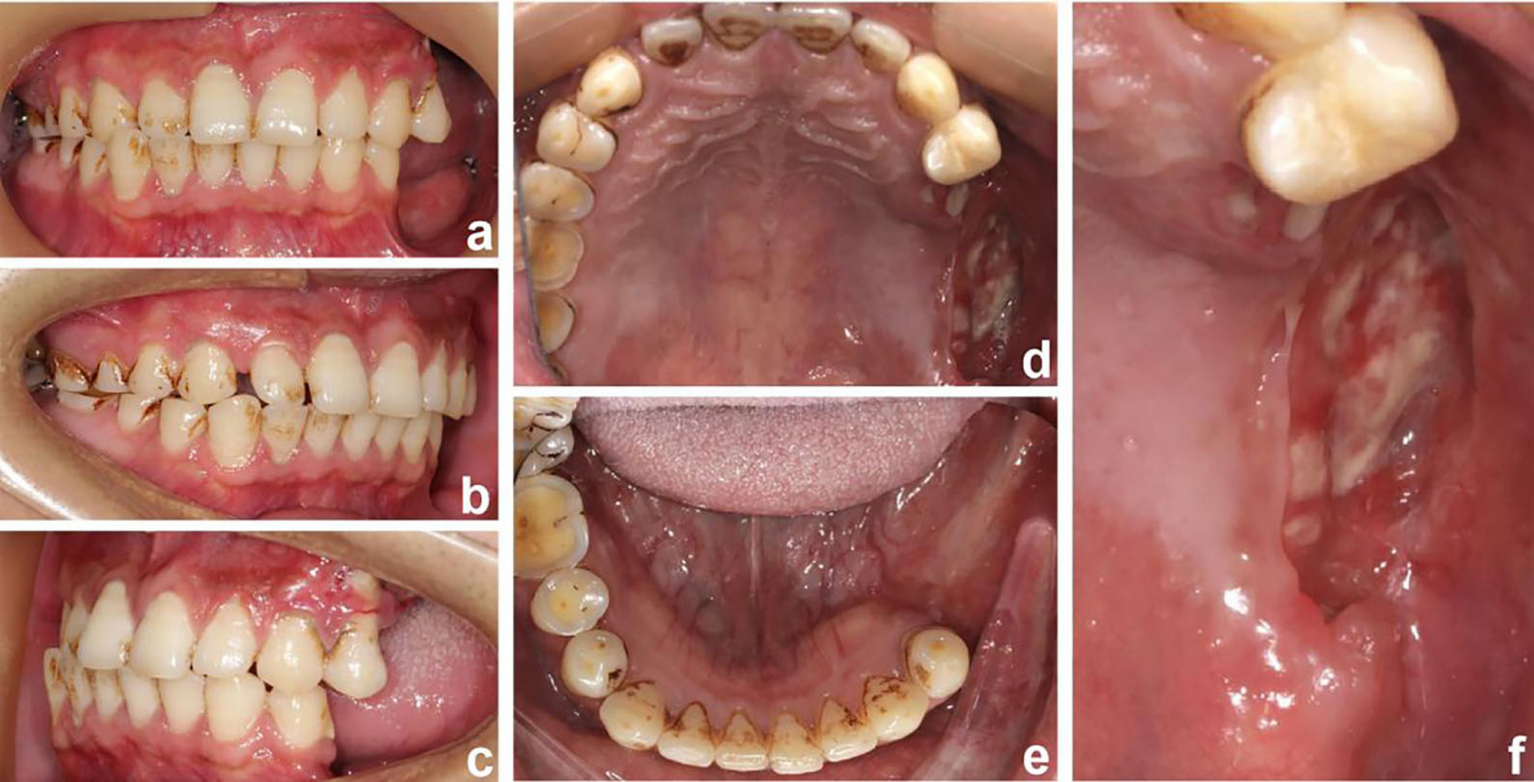
Pretreatment intraoral photographs showing (a) frontal occlusion, (b) right occlusion, (c) left occlusion, (d) maxillary occlusion, (e) mandibular occlusion, and (f) dehiscent gingiva.

**Figure 2:**
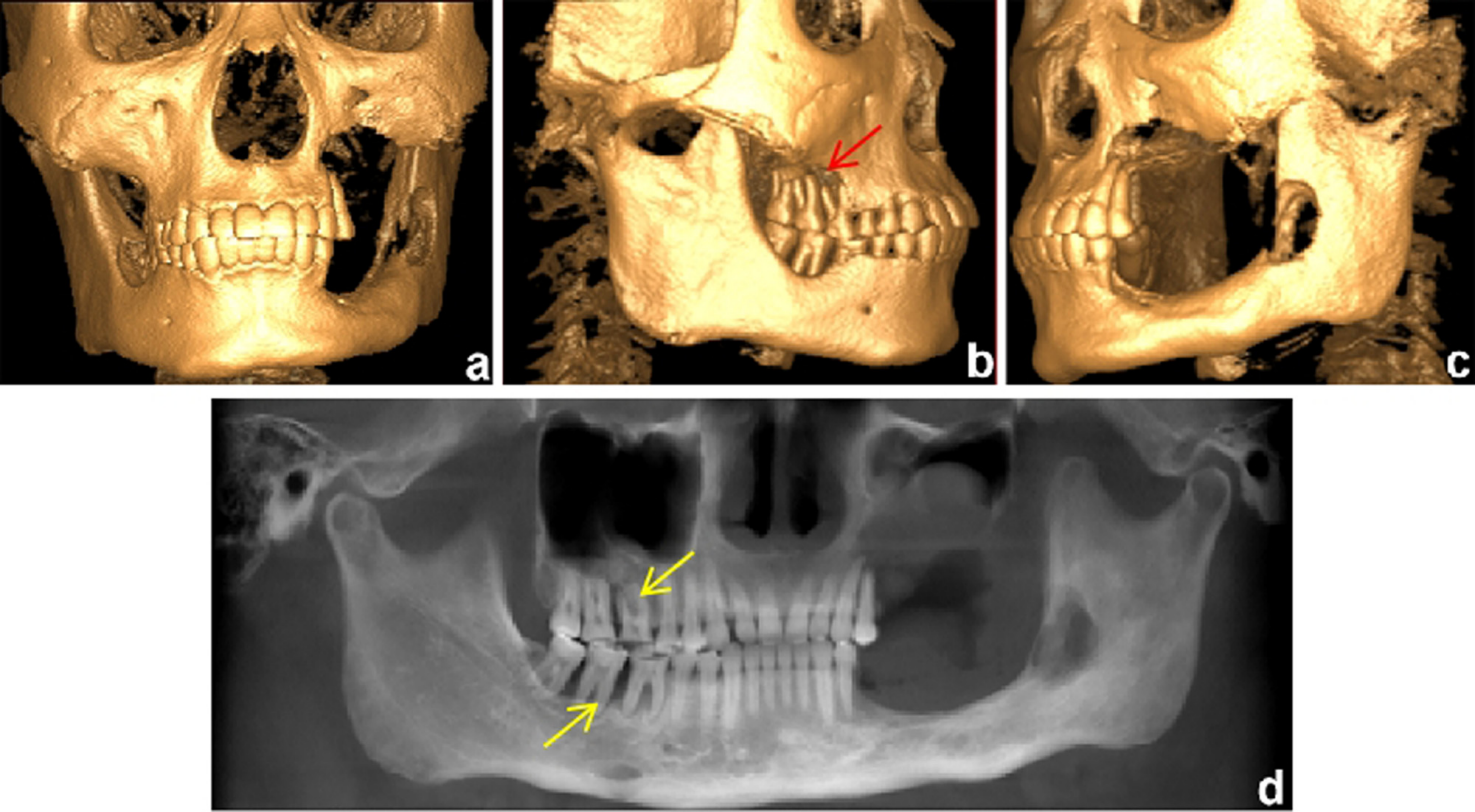
Pretreatment 3D-reconstruction images showing severe bone destruction of (a) maxillary sinus, (b) maxilla (red array), and (c) mandible. The baseline CBCT shows (d) the characteristic “floating teeth” appearance (yellow array).

A 27-year-old Chinese man presented with progressive mobility of the posterior teeth. Symptoms began 6 years back with the left mandibular first molar, followed by consecutive loss of the left posterior teeth at a rate of one tooth per year. Despite undergoing periodontal treatment, the patient experienced no improvement in tooth mobility. Two months prior to diagnosis, the patient experienced gingival swelling in the left maxillary posterior region, accompanied by severe pain. Intraoral examination revealed complete tooth loss from teeth 25–28 and teeth 35–38, along with diffuse mucosal erosion in the upper left posterior area. Extraoral examination identified multiple painless and movable enlarged lymph nodes bilaterally in the submandibular region (Figure 1). The patient had a 10-year history of smoking and had been chewing betel nuts for 9 years. Cone-beam computed tomography (CT) revealed a poorly demarcated radiolucent area on the left mandibular ramus and complete resorption of the alveolar ridge in regions centred on teeth 25–28. Additionally, extensive bone loss was observed around teeth 16, 46, and 47, resulting in a “floating-in-the-air” appearance of these teeth (Figure 2).

**Figure 3:**
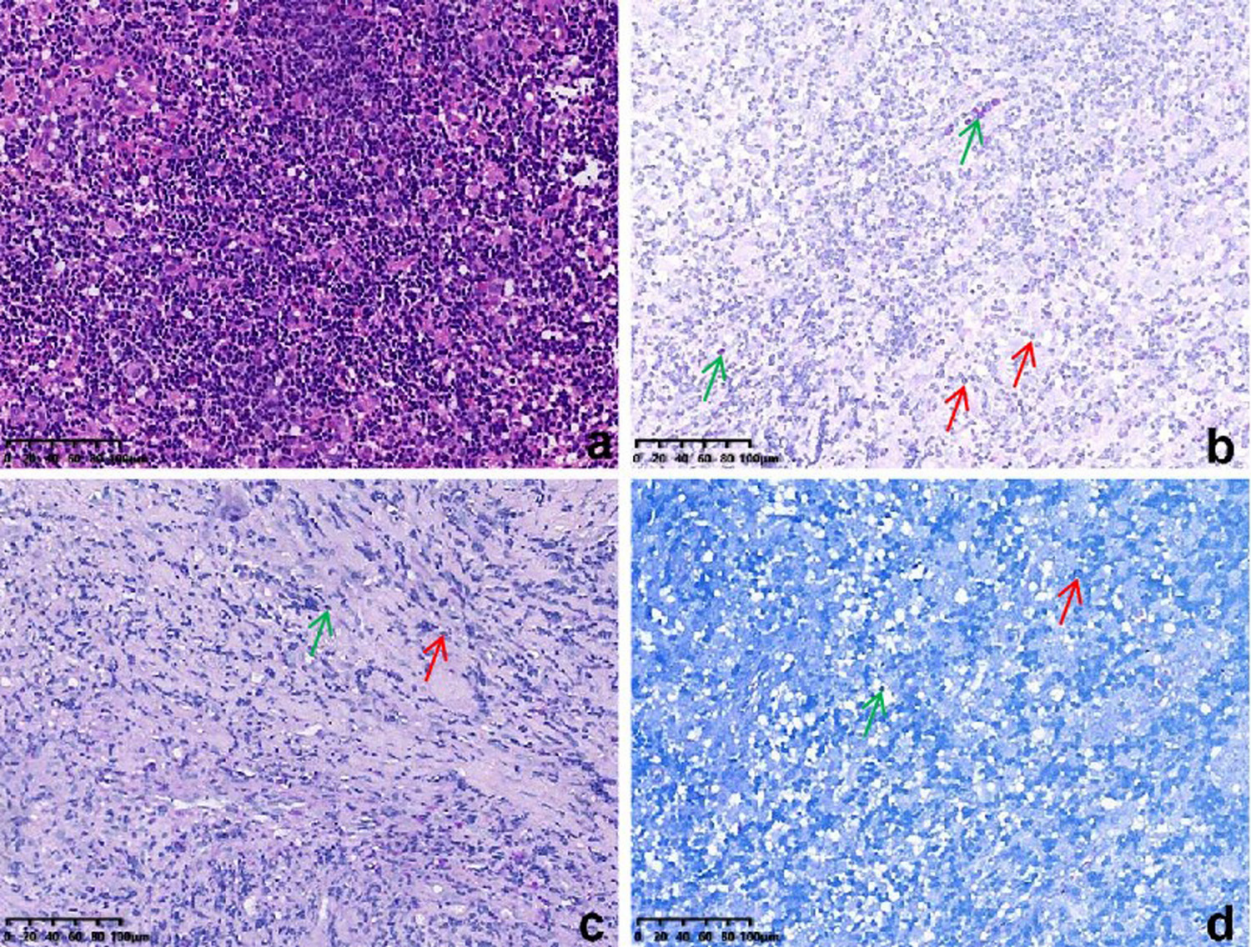
Photomicrographs showing (a) histiocytic infiltrates (Hematoxylin and Eosin stain), (b) PAS stain (−), (c) digestive PAS stain (−), and (d) acid-fast stain (−). The green arrows represent eosinophilic cells, and red arrows represent Langerhans cells.

**Figure 4:**
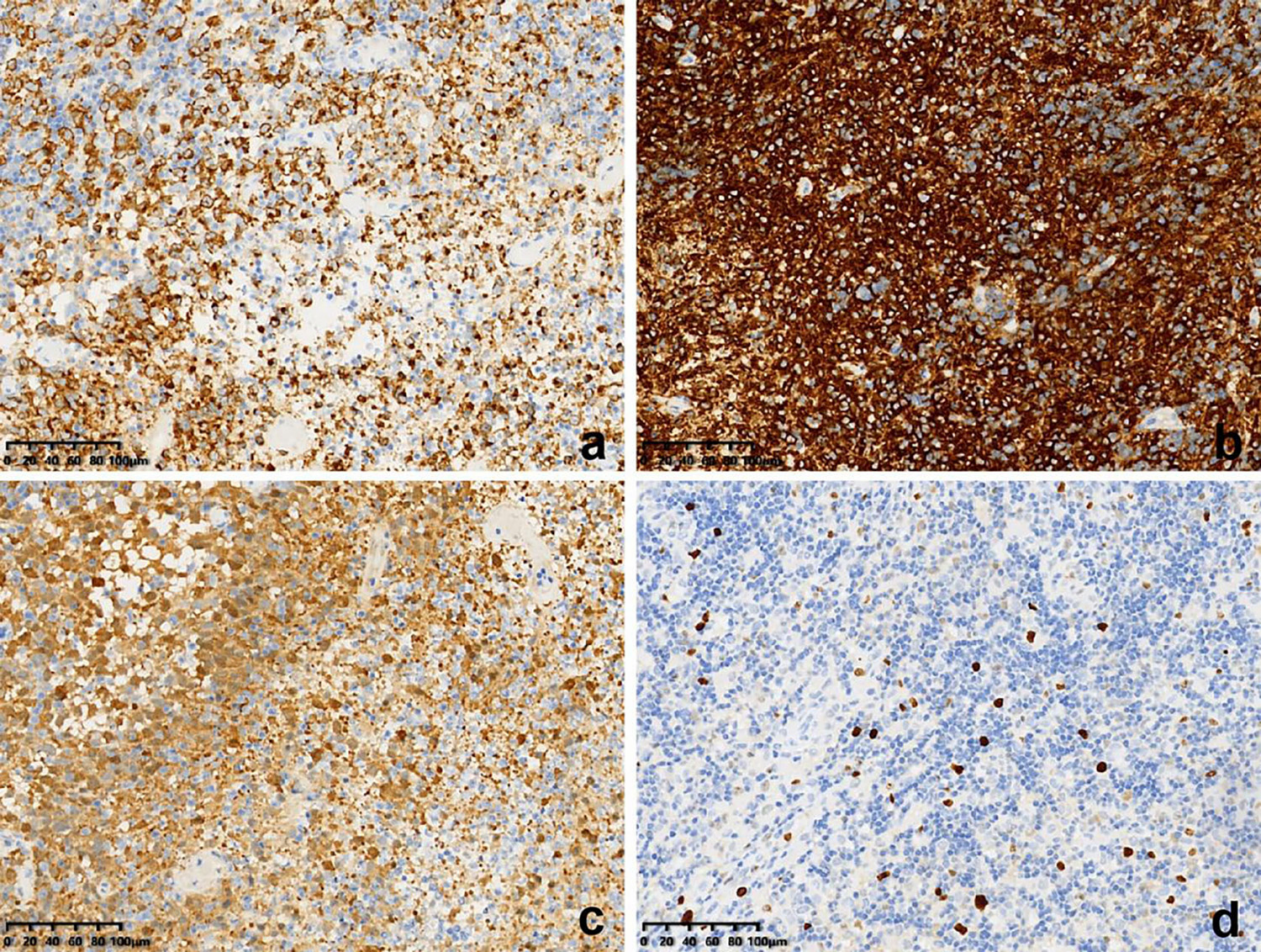
Immunohistochemical staining showing reactivity for (a) Langerin, (b) CD1a, (c) S-100, and (d) a positive index of 20% for Ki67.

**Figure 5:**
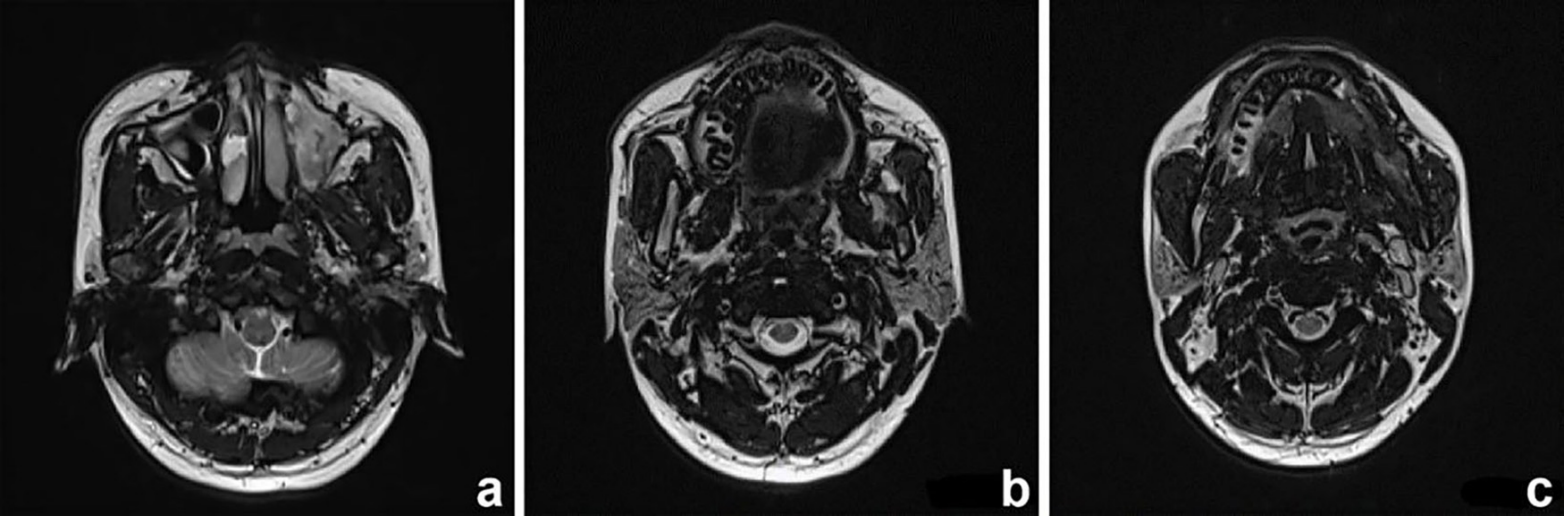
MRI scans showing (a) maxillary sinus, (b) maxillary, and (c) mandibular involvement.

**Figure 6:**
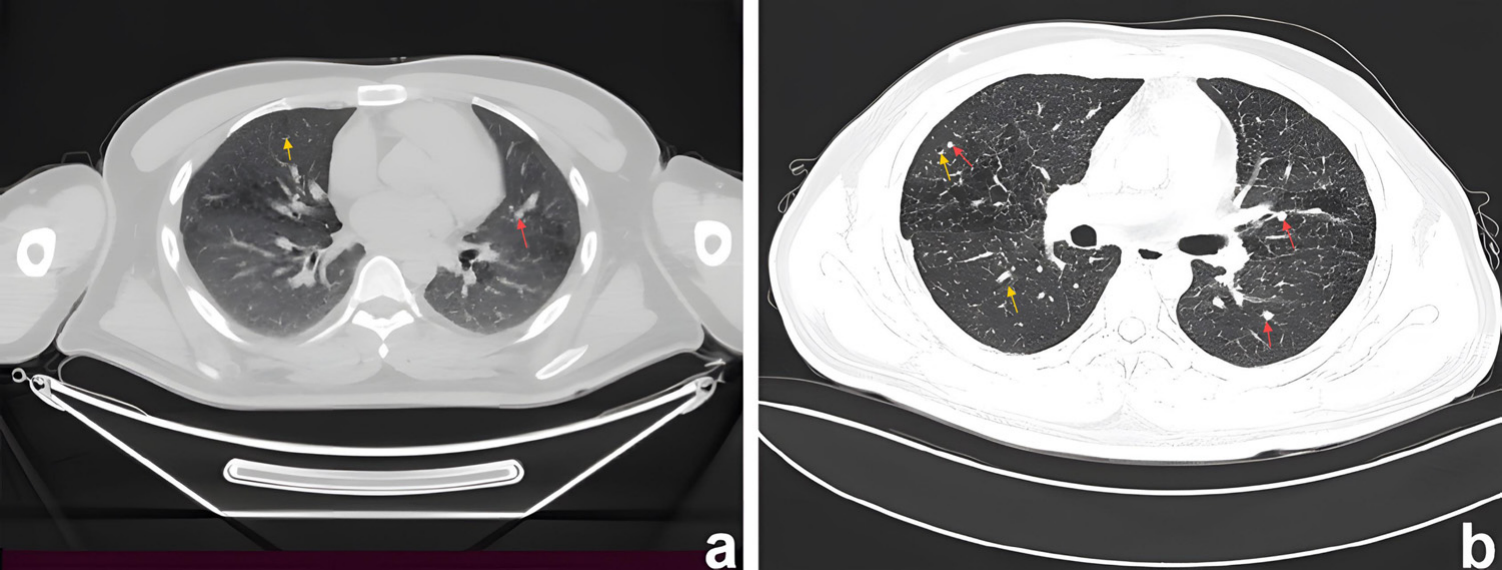
Computed tomography of chest showing nodules (red arrows)/cyst (yellow arrows) in both lungs. (a) CT axial view shows cysts of varying sizes in the upper lobes of both lungs. (b) Thin-section CT showed nodules with unclear boundaries, mainly located in the middle lobes of both lungs. Some nodules had clear centres, indicating that they would become cystic.


Samples collected from the eroded mucosa in the upper left posterior tooth area underwent histological and immunohistochemical examination using an automated immunohistochemistry stainer (Ventana Benchmark XT; Ventana Medical Systems, Tucson, AZ, USA). Histological analysis revealed dense infiltrates of Langerhans cells interspersed with eosinophilic inflammatory cells (Figure 3a). Histochemical staining revealed no evidence of malignancy or tuberculosis infection (Figure 3b–d). Immunohistochemically, the Langerhans cells exhibited immunoreactivity to CD1a (mouse monoclonal anti-CD1a antibody; MBL Beijing Biotech Co., Ltd, Beijing, China), Langerin (mouse monoclonal anti-Langerin antibody; MBL Beijing Biotech Co., Ltd) and S100 proteins (rabbit polyclonal antiS100 protein antibody; Zymed Laboratories, San Francisco, CA, USA). The Ki67 (mouse monoclonal anti-Ki67 antibody; MBL Beijing Biotech Co., Ltd) positive index was 20% (Figure 4). Oral and neck magnetic resonance imaging (MRI) scans depicted T2-weighted hyperintense and inhomogeneous contrast-enhancing lesions with a width of up to 0.5 cm in the left posterior maxilla (Figure 5). High-resolution CT (HRCT) examination revealed multiple cysts and nodules in both lungs (Figure 6). Combining the histological analysis of eroded mucosa and HRCT, the patient was diagnosed with LCH involving oral and pulmonary regions. The patient received eight cycles of vinblastine–etoposide–prednisone chemotherapy (4 mg of vinblastine on day 1, 300 mg of etoposide on day 1, 10 mg of prednisone on days 1–5). After systemic chemotherapy, the patient was lost to follow-up.

**Informed consent:** Informed consent has been obtained from all individuals included in this study.
**Ethical approval:** The research related to human use has been complied with all the relevant national regulations, institutional policies and in accordance with the tenets of the Helsinki Declaration, and has been approved by the authors’ institutional review board or equivalent committee.

## 3 Discussion

The clinical manifestations of LCH are diverse. Pulmonary involvement is observed in up to 40% of patients, typically presenting as nodular or reticular patterns on chest radiographs. As a result, chest CT scans are often pivotal in diagnosis. The formation of early-stage granulomas in the lung is easy to observe. However, detecting nodule formation in solid organs is challenging through imaging, despite the lesions’ similar nature [10]. Therefore, the diagnosis of LCH requires a comprehensive approach, integrating clinical, radiological, pathological, and immunophenotypic analyses. Among these, the immunophenotypic detection of CD1a and CD207 expression in lesional histiocytes is of paramount importance [11].

LCH rarely affects oral regions, often presenting with symptoms resembling periodontal disease, lymphoma, or osteomyelitis, resulting in misdiagnosis and delayed treatment [12]. Periodontal diseases clinically manifest as inflammation of the gums, leading to gum redness, swelling, bleeding, bad breath, receding gums, and loose teeth. Similarly, periodontal manifestations of LCH include periodontal pockets, recession, furcation involvement, gingival bleeding, and tooth mobility. In this case, the patient experienced excessive molar mobility and tooth loss, initially resembling chronic periodontitis. However, conventional periodontal treatment proved ineffective, and the bone destruction extended beyond the alveolar bone, involving the left mandibular ramus and the interior walls of the left maxillary sinus. Given the rapid progression of periodontal destruction, particularly in a relatively young patient, suspicion of systemic factors was warranted, prompting a biopsy and immunohistochemical test, which confirmed LCH. Further CT and MRI scans revealed involvement in the jaws and lungs.

Although treatment modalities for paediatric LCH have been extensively described, no gold standard exists for adult LCH treatment. Various regimens have been used, encompassing surgical excision, radiotherapy, systemic chemotherapy, and targeted therapy. Clinical evidence demonstrates that the 3-year overall survival is 94.4% for SS-LCH and 54.7% for MS-LCH in adults [13]. For patients with SS-LCH, surgical excision and radiotherapy are considered curative [14]. Low-risk patients with MS-LCH are typically managed with vinblastine and prednisone [8], whereas high-risk patients with MS-LCH may benefit from mercaptopurine, vinblastine, and prednisone [15]. A previous study determined that intense treatment with 6-mercaptopurine followed by prednisone and vinblastine significantly increased the response and reduced mortality in MS-LCH with the risk of organ involvement [16]. Additionally, a study indicated that the involvement of organs and being over 50 years predicted a less favourable outcome. However, receiving cytarabine-based therapy has had favourable outcomes, suggesting that a cytarabine-based regimen could be considered a first-line treatment for adult patients with LCH [13].

Targeted therapy represents a promising emerging approach to treatment. Additionally, BRAF inhibitors (e.g., vemurafenib and dabrafenib) have exhibited efficacy in refractory MS-LCH. However, their use is associated with severe side effects, including squamous cell carcinoma and lesion relapse. In cases with extensive maxillofacial involvement, as in this case, surgery is not the primary option because of the potential negative impacts on masticatory function, aesthetics, and the complexity of reconstructing the stomatognathic system. Once oral lesions achieve remission and periodontal conditions stabilize, post-systemic chemotherapy, dental treatments such as implant-supported prostheses, or partial dentures become appropriate.

## 4 Conclusion

In this case, LCH remained unrecognized and untreated for 6 years, leading to rapid jawbone destruction, tooth loss and subsequent lung involvement. Early recognition and a multidisciplinary treatment approach are crucial. Further research and clinical guidelines are needed to manage adult-onset LCH effectively.
